# Supplementary material for: A micro RNA mediates shoot control of root branching
Source: Nat Commun. 2023 Dec 6;14:8083. doi: 10.1038/s41467-023-43738-6 (PMC10700597; doi:10.1038/s41467-023-43738-6)
Supplement: Supplementary file 1 — Supplementary Information [file 41467_2023_43738_MOESM1_ESM.pdf]

A micro RNA mediates shoot control of root branching

SUPPLEMENTARY INFORMATION

Moritz Sexauer[1,4,5], Hemal Bhasin[1,2,5], Maria Schön[1], Elena Roitsch[1,3], Caroline Wall[1], Ulrike Herzog[1] and Katharina Markmann[1,3,4] *

Supplementary Tables

Supplementary Table 1. Relative transcript abundances of *MIR21111a/b* and *HOLT* in *A. thaliana* tissues. Data retrieved from RNAseq based developmental transcript profiling using two biological replicates[1]. Numbers represent relative expression values, with the maximum normalized read number observed for a particular locus set =1. Only an excerpt of the original dataset is represented. Expression foci are in bold.

	MIR2111a (AT3G09285)	***MIR2111b (AT5G02035)***	***HOLT (AT3G27150)***
***root** apex*	0	0	**0.5**
***root** without apex*	0	0	**1**
mature leaf, blade	0	0.11	0.01
*mature leaf, **petiole***	**0.45**	0.11	0
*mature leaf, **vein***	0	**0.21**	0
senescent leaf, petiole	0	0.13	0
*senescent leaf, **vein***	**1**	**0.36**	0.01
dry seeds	0	**1**	0

Supplementary Table 2. Oligonucleotide sequences used in cloning and genotyping.

**Target**	**Orientation**	**Sequence (5'-3')**
Gateway cloning of *At MIR2111b* overexpression fragment	forward	GGGGACAAGTTTGTACAAAAAAGCAGGCTCATAACCTTTCCCTCTCATG
Gateway cloning of *At MIR2111b* overexpression fragment	reverse	GGGGACCACTTTGTACAAGAAAGCTGGGTTGGTTTTCGTTAACCAAAGG
Gateway cloning of *At MIR2111a* promoter fragment	forward	GGGGACAAGTTTGTACAAAAAAGCAGGCTGTTAATGGAGTTATTTATAG
Gateway cloning of *At MIR2111a* promoter fragment	reverse	GGGGACCACTTTGTACAAGAAAGCTGGGTTCACCCCCCATAATGCGCGC
Gateway cloning of *At MIR2111b* promoter fragment	forward	GGGGACAAGTTTGTACAAAAAAGCAGGCTCATAACCTTTCCCTCTCATG
Gateway cloning of *At MIR2111b* promoter fragment	reverse	GGGGACCACTTTGTACAAGAAAGCTGGGTTTGTTTTATGTTAATGGTGA
Genotyping holt-1	forward	CTTCCAAGAGTTTGCATTTGC
Genotyping holt-1	reverse	ATCAAATCGGTAGAATTGGGG
Genotyping holt-2	forward	TTTTTAGCCTCTCAAGAATTTGG
Genotyping holt-2	reverse	TAGCTGGTGGGTTGAAAATTG
Genotyping holt-1/2 insertion	reverse	ATTTTGCCGATTTCGGAAC

Supplementary Table 3. Oligonucleotide sequences used in reverse transcription. Nucleotides complementary or reverse complementary to small RNA sequences are in bold.

**Target**	**Orientation**	**Sequence (5'-3')**
PolyA signal	reverse	TTTTTTTTTTTTTTTTTV
Lj miR2111a/b	reverse	GTCGTATCCAGTGCAGGGTCCGAGGTATTCGCACTGGATACGAC**TACACC**
At miR2111	reverse	GTCGTATCCAGTGCAGGGTCCGAGGTATTCGCACTGGATACGAC**TAAACC**
AtHOLT	reverse	TAACCCACAATAGCACCGT
AtUBQ2	reverse	CAGATGAATAATGGGGCTC
AtPP2A	reverse	CGCCCAACGAACAAATCACA
U6	reverse	GTGCAGGGTCCGAGGTTTTGGACCATTTCTCGAT

Supplementary Table 4. Oligonucleotide sequences used in quantitative PCR. Nucleotides complementary or reverse complementary to small RNA sequences are in bold.

**Target**	**Orientation**	**Sequence (5'-3')**
miR2111a/b stemloop primers	reverse	AGTGCAGGGTCCGAGGTATTC
Lj miR2111a/b and *At* miR2111	forward	GCGCG**TAATCTGCATCCTGAG**
LjTML	forward	GCCAACAATTGCCTGAAACCAGATG
	reverse	CTTTATGGTGTTTCTCTCTATGAATGCTG
LjPP2a	forward	GTAAATGCGTCTAAAGATAGGGTCC
	reverse	ACTAGACTGTAGTGCTTGAGAGGC
LjATPs	forward	CAATGTCGCCAAGGCCCATGGTG
	reverse	AACACCACTCTCGATCATTTCTCTG
AtPP2A	forward	GGTAATAACTGCATCTAAAGACAGAGTTCC
	reverse	CCACAACCGCTTGGTCG
AtUBQ2	forward	CCAAGATCCAGGACAAAGAAGGA
	reverse	TGGAGACGAGCATAACACTTGC
AtHOLT	forward	TTCTGTCCAATCTCCACCGCTC
	reverse	ACTTAAACGCAACGCCCCATC
AtNRT1.1	forward	GAGAGGCTGACGACGTTAGGT
	reverse	ATTGCGGCGAATATAGCAATCG
AtNRT1.5	forward	CGCAAAATGTCTTGCCTAGAG
	reverse	CATAGTAATCCACTGTCCCATCTCTT
AtNRT2.1	forward	GAAATCGAGCTACCTTGGAGAA
	reverse	TTGTAACGGCATACCACAGAA
AtNRT3.1	forward	TGGGTCTAAAAGAGAGGCTGA
	reverse	GCCATGGTCGGTCAACTT
αTubulin	forward	GGTTCGACAACAGCGGTAGA
	reverse	GTGGCACCGGATCAGGATTT
U6	forward	GGAACGATACAGAGAAGATTAGCA
	reverse	GTGCAGGGTCCGAGGT

Supplementary Table 5. Oligonucleotide sequences used in mutant characterization.

Target	Orientation	Sequence (5'-3')
HOLT 3' of holt-1 insertion	forward	ATGTTGACGCTAGGAGAAGA
	reverse	ATAGTCCGCGTCTTTCGGCTTCAAACATGTACTGCTCAGAGCCTTAG
HOLT full length	forward	ATGTTGACGCTAGGAGAAGA
	reverse	TTACGCAATCATTACACAACAG

Supplementary Figures

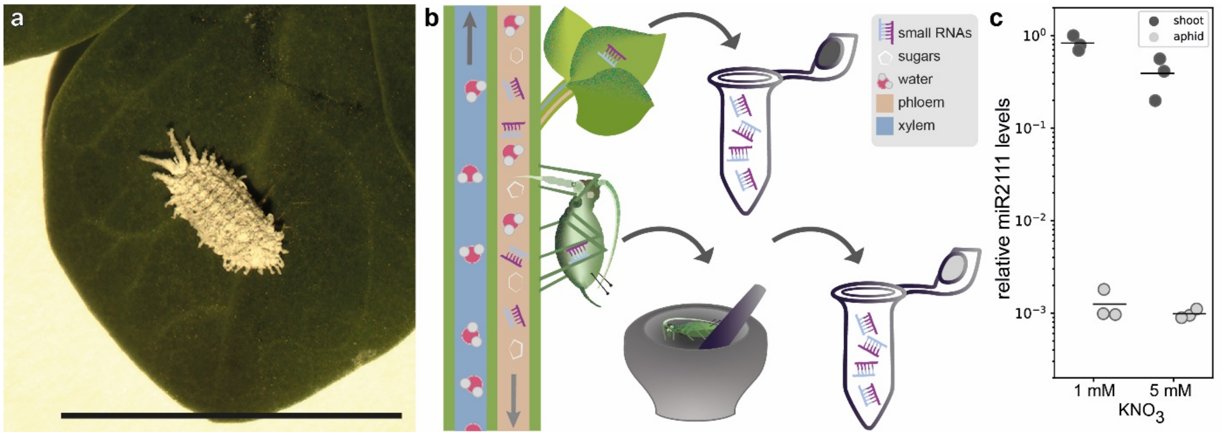

Supplementary Fig. 1. Mature miR2111 can be traced in aphids feeding on *L. japonicus* (Lotus) phloem sap. **a** *Planococcus citri* on Lotus leaf, scalebar equals 1 cm. **b** Simplified sketch of experimental procedure of an aphid feeding experiment. Plants are grown for four weeks in absence of aphids, then colonized by a small aphid population, which propagates for two weeks while feeding on phloem sap. During feeding, aphids ingest mobile small RNAs, which have been suggested to travel through the phloem as mature duplices[2]. Possible association of miRNAs with proteins during phloem transfer is not considered here. Aphids and plant material are harvested separately for RNA extraction and qRT-PCR based detection of mature plant miR2111. **c** miR2111 levels in shoot tissue of Lotus ecotype Gifu wild type plants and aphids (*Planococcus citri*) after feeding on respective plants grown at indicated nitrate conditions. qRT-PCR analyses. miR2111 levels from Lotus shoot RNA extracts are relative to two endogenous reference genes, miR2111 levels from aphid RNA extracts are relative to one aphid reference gene. Host and aphid tissues were harvested after two weeks of aphid feeding and six weeks after plant germination. Sample size, replicates and exact p-values are listed in Supplementary Data 1. Dotplots show individual data points and a line indicating their average value.

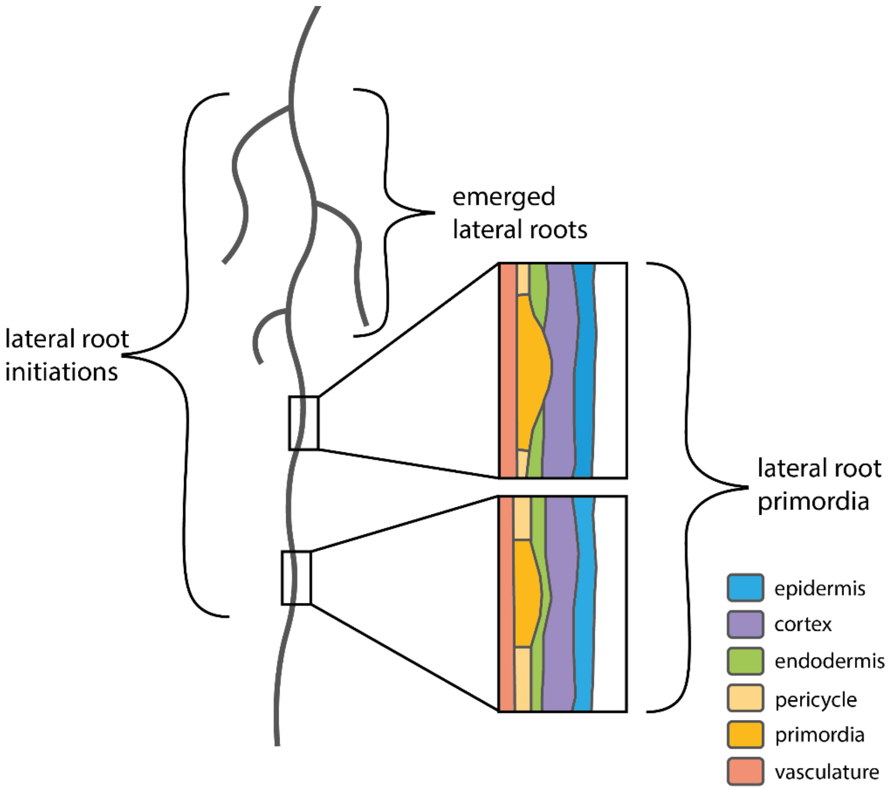

Supplementary Fig. 2. Schematic representation of lateral root initiations encompassing both emerged lateral roots and lateral root primordia. Insets show schematic representation of lateral root primordia, with different colors indicating root cell types as indicated.

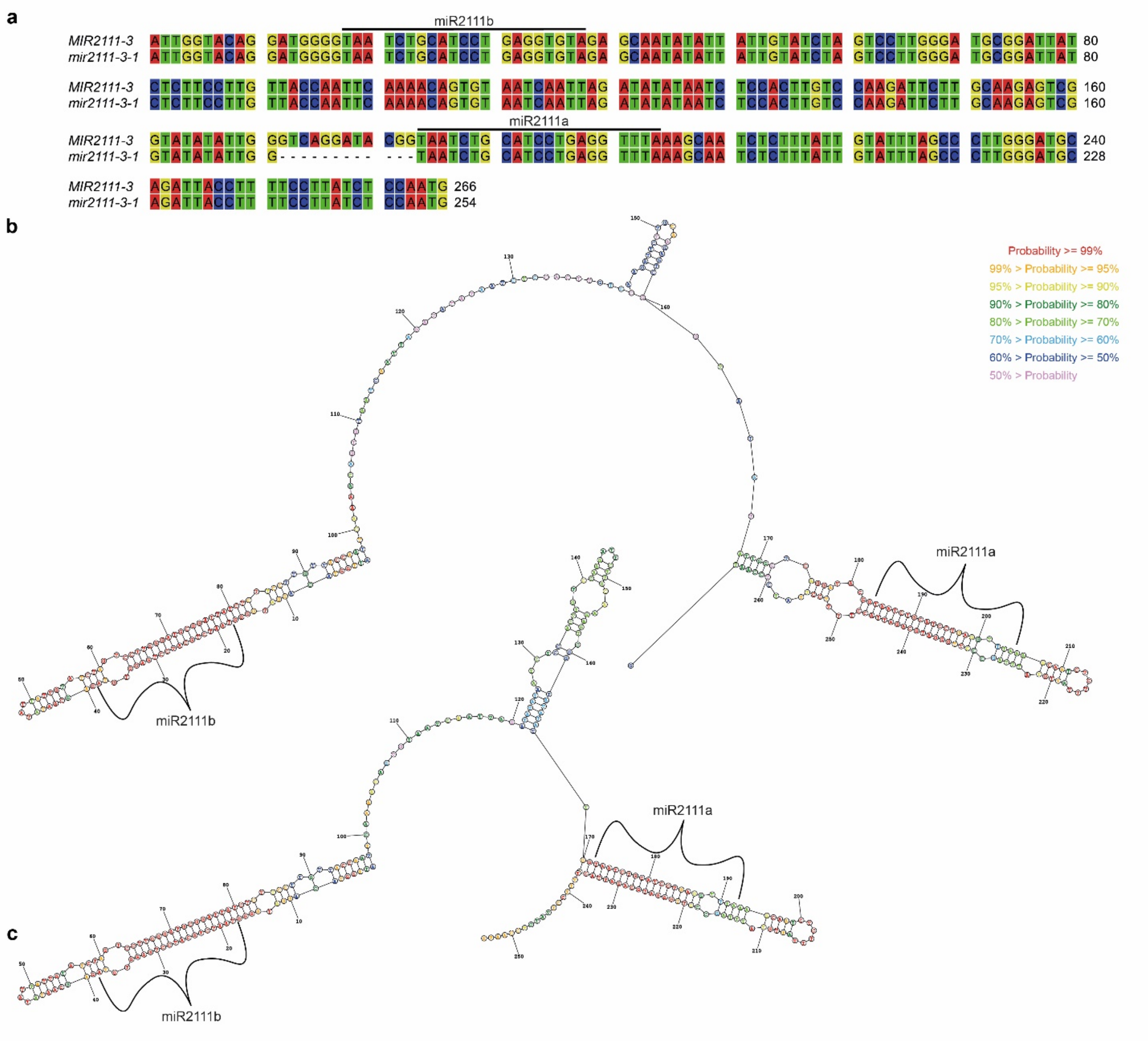

Supplementary Fig. 3. Characterization of the *L. japonicus MIR2111-3* CRISPR/Cas9-mediated knockout deletion. a Alignment of the *MIR2111-3* sequence and the 12 bp deletion in the *mir2111-3-1* allele. **b-c** Predicted hairpin structures of *MIR2111-3* without (**b**) and with the deletion indicated in **a** (**c**). **b-c** Secondary structure was modelled using the MaxExpect algorithm with default settings[3].

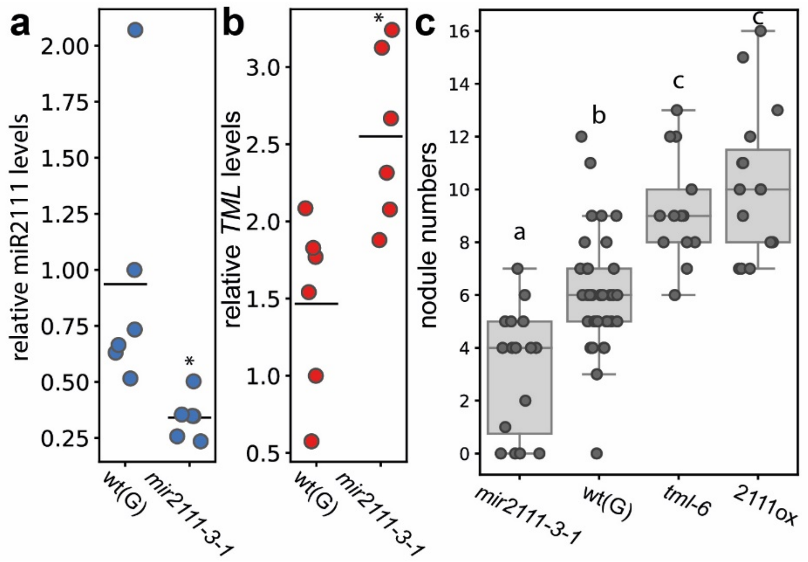

Supplementary Fig. 4. Alterations of miR2111 and *TML* levels influence nodule numbers in *L. japonicus* (Lotus). a, b miR2111 (**a**) and *TML* levels (**b**) in roots of Lotus ecotype Gifu wild type (wt(G)) and *mir2111-3-1* plants. **c** Nodule numbers of wt(G), *tml-6*, *MIR2111-3* ox (2111ox) and *mir2111-3-1* mutants, *tml-6* and 2111ox plants. **a-b** qRT-PCR analyses. Root RNA levels are relative to those of two reference genes. Tissue was harvested 14 days after transfer. Plants were grown at 5 mM nitrate. **a-c** Comparisons used Student's *t*-test (*=p<0.05) (**a-b**) or (**c**) analysis of variance (ANOVA) and post-hoc Tukey testing (p≤0.05), with distinct letters indicating significant differences. **c** plants evaluated three weeks after inoculation with *M. loti*, plants grown at 0 mM nitrate. Sample size, replicates and exact p-values are listed in Supplementary Data 1. Dotplots show individual data points and a line indicating their average value. Boxplot central line shows median value, box limits indicate the 25th and 75th percentile. Whiskers extend 1.5 times the interquartile range, or to the last datapoint. Individual datapoints are represented by dots.

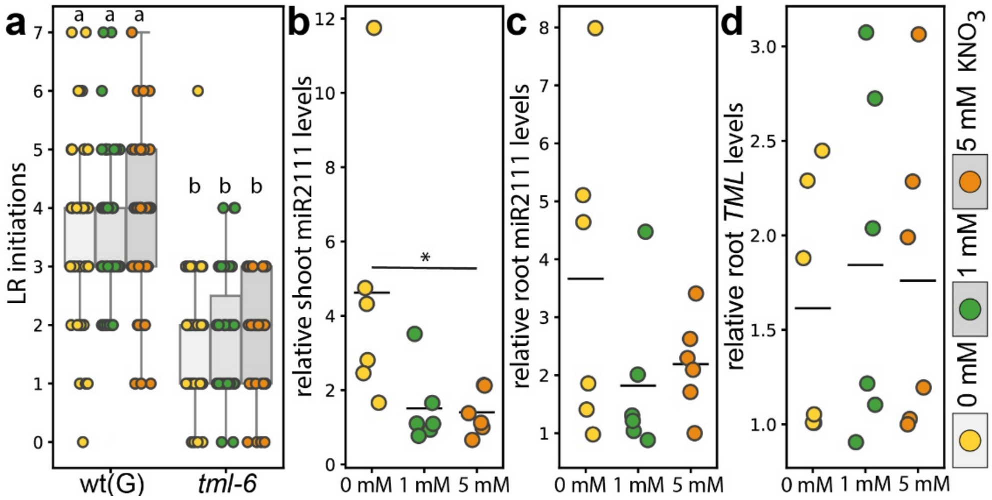

Supplementary Fig. 5. *L. japonicus* (Lotus) ecotype Gifu lateral root (LR) initiation numbers and *TML* levels are nitrate independent. a LR initiations in wild type Gifu (wt(G)) and *tml-6* plants 10 days post transfer. **b-d** Relative mature miR2111 levels in shoots (**b)** and roots (**c**) and relative *TML* levels in the same root systems (**d**) of ecotype Gifu B-129 wild type plants after 14 days of cultivation. **b-d** qRT-PCR analyses. RNA levels are relative to those of two reference genes. Comparisons used analysis of variance (ANOVA) and post-hoc Tukey testing ($p \leq 0.05$), with distinct letters indicating significant differences (**a**) or Student's t-test (*=$p<0.05$) (**b**). Sample size, replicates and exact p-values are listed in Supplementary Data 1. Dotplots show individual data points and a line indicating their average value. Boxplot central line shows median value, box limits indicate the 25th and 75th percentile. Whiskers extend 1.5 times the interquartile range, or to the last datapoint. Individual datapoints are represented by dots.

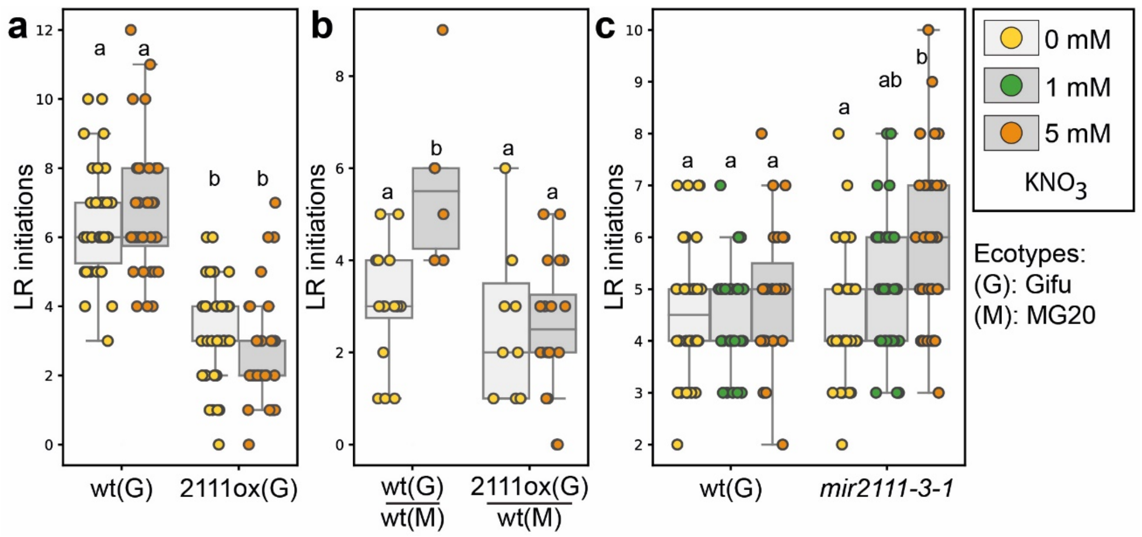

Supplementary Fig. 6. Low miR2111 levels are required and sufficient for enhanced lateral root (LR) initiation under high nitrate conditions in *L. japonicus* (Lotus). a-c LR initiations of Lotus ecotype Gifu wild type (wt(G)) and *mir2111-3-1* plants (**a**), heterografted plants assembled of Lotus ecotype MG20 wild type (wt(M)) root stocks and ecotype Gifu wild type or *MIR2111-3* overexpression (2111ox) shoots (**b**), and ecotype Gifu wild type and *mir2111-3-1* plants (**c**). **a**-**b** Plants were grown at indicated nitrate concentrations and analyzed after two weeks of cultivation (**a, b**) or graft regeneration (**c**), respectively. Comparisons used analysis of variance (ANOVA) and post-hoc Tukey testing ($p \leq 0.05$), with distinct letters indicating significant differences. Sample size, replicates and exact p-values are listed in Supplementary Data 1. Boxplot central line shows median value, box limits indicate the 25th and 75th percentile. Whiskers extend 1.5 times the interquartile range, or to the last datapoint. Individual datapoints are represented by dots.

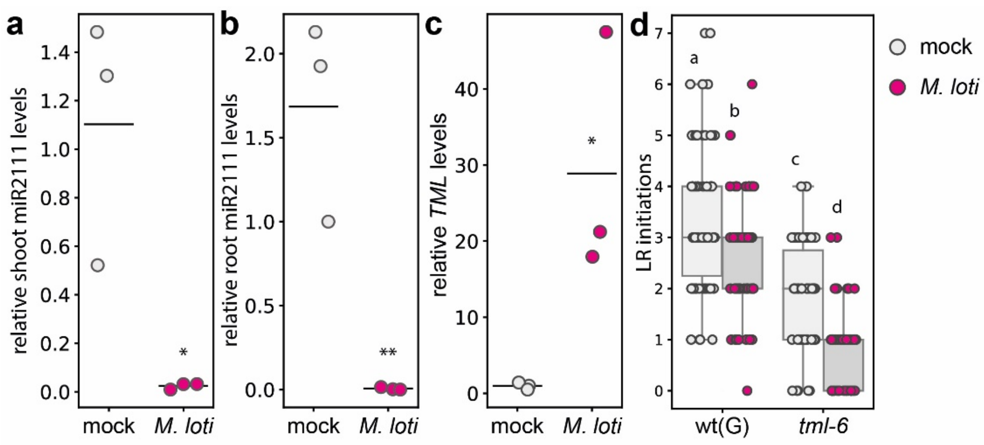

Supplementary Fig. 7. Symbiosis restricts lateral root (LR) initiation in a *TML*-independent manner in *L. japonicus* (Lotus). a-c Relative mature miR2111 levels in shoots (**a**) and roots (**b**) and relative *TML* levels (**c**) in the same Lotus plants (ecotype Gifu wild type (wt(G))) at 21 days post inoculation. Plant were cultivated for 3 weeks. **d** LR initiations in symbiotic and non-symbiotic wild type and *tml-6* plants at 10 days post inoculation. **a**-**c** qRT-PCR analyses. RNA levels are relative to those of two reference genes. Plants were inoculated with *Mesorhizobium loti*, or treated with water (mock control). Comparisons used analysis of variance (ANOVA) and post-hoc Tukey testing ($p \leq 0.05$) (**d**), with distinct letters indicating significant differences or Student's *t*-test (*=$p<0.05$) (**a-c**). Sample size, replicates and exact p-values are listed in Supplementary Data 1. Dotplots show individual data points and a line indicating their average value. Boxplot central line shows median value, box limits indicate the 25th and 75th percentile. Whiskers extend 1.5 times the interquartile range, or to the last datapoint. Individual datapoints are represented by dots.

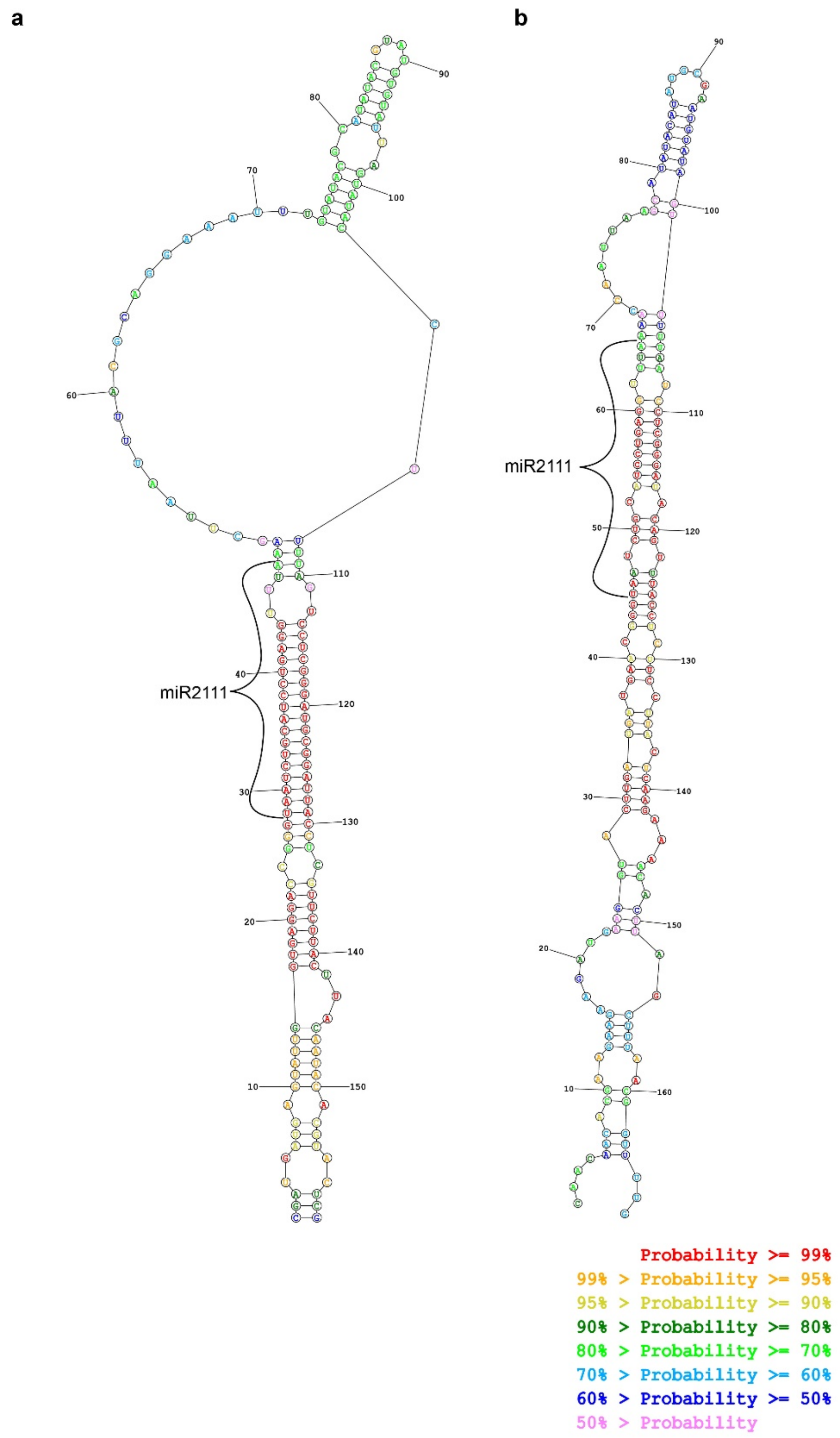

Supplementary Fig. 8. The *A. thaliana* genome contains one miR2111 isoform at two distinct loci. a-b, *MIR2111a* (**a**) and *MIR2111b* (**b**) transcripts are predicted to form hairpin structures. Secondary structure was modelled using the MaxExpect algorithm with default settings[3].

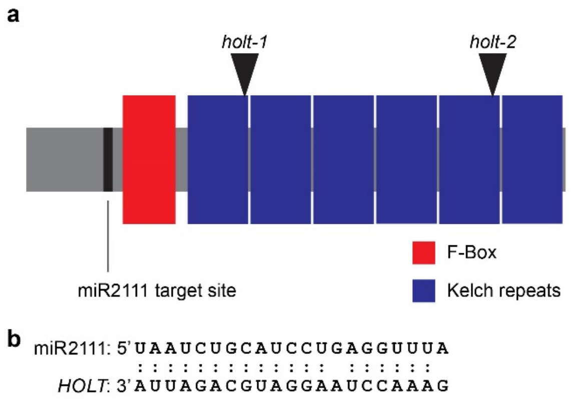

Supplementary Fig. 9. *A. thaliana HOLT* is an F-Box Kelch Repeat protein with a conserved miR2111 complementary site. a *HOLT* gene and predicted protein features including the miR2111 target site as indicated, and position of the t-DNA insertion in *holt-1*and *holt-2* (black arrowheads). The t-DNA insertion positions were confirmed by Sanger sequencing. Red and blue boxes indicate predicted protein domains as indicated. **b** Alignment of miR2111 and *HOLT* mRNA at the target site. Colons indicate A/U and G/C base pairing.

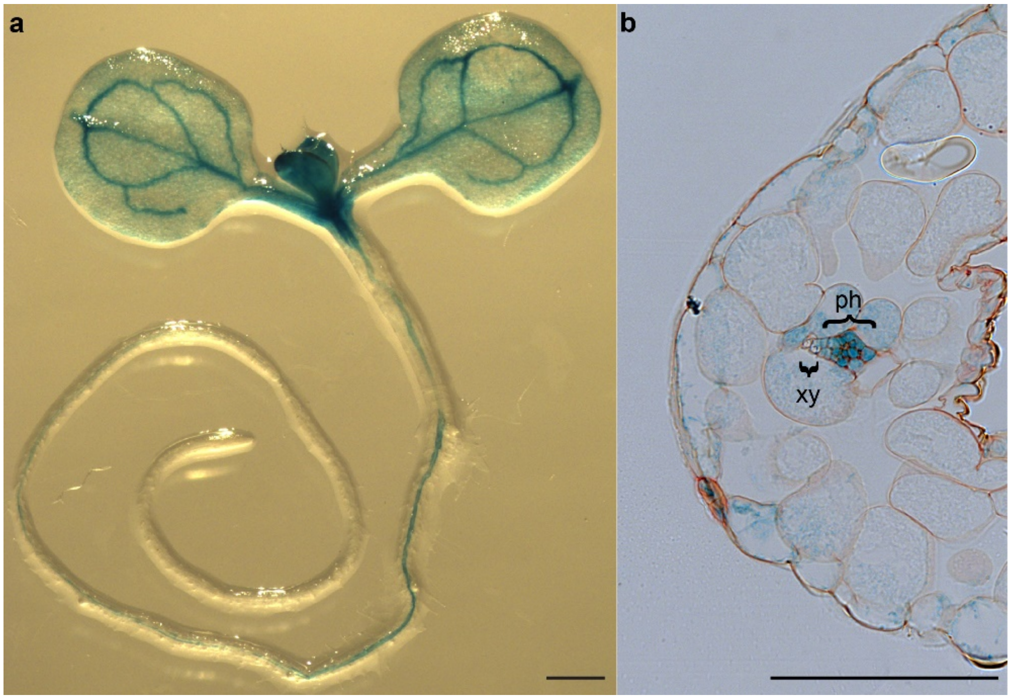

Supplementary Fig. 10. *A. thaliana MIR2111a* is predominantly expressed in leaf vein phloem.

a Five day-old stably transformed *A. thaliana* seedlings expressing *pMIR2111a:GUS* show GUS activity in leaf veins and root vasculature. **b** *GUS* expression is strongest in leaf phloem. **a**,**b** Scale bar equals 500 µm (**a**) or 100 µm (**b**). **a-b** Analysis of three independent lines showed similar results. All 34 tested plants of 3 independent lines showed a likewise expression pattern.

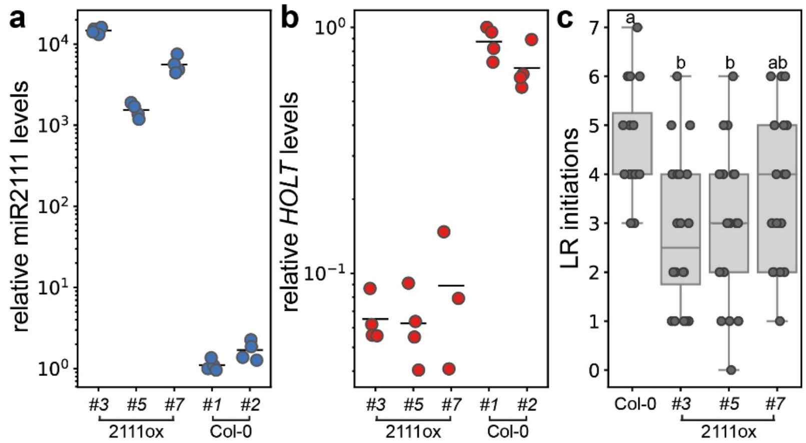

Supplementary Fig. 11. ***A. thaliana p35s::MIR2111b*** **(2111ox) lines show strongly reduced** ***HOLT*** **levels compared to wild type plants. a-c** miR2111 (**a**) and *HOLT* (**b**) levels and lateral root initiation (**c**) of independent 2111ox lines. Line #3 was selected for further propagation and experiments. **c** Plants were grown for seven days on 1 mM nitrate. **a**, **b** qRT-PCR analyses. RNA levels are relative to those of two reference genes (**b**) or relative to U6 (**a**). **c** Comparisons used analysis of variance (ANOVA) and post-hoc Tukey testing ($p \leq 0.05$). Sample size, replicates and exact p-values are listed in Supplementary Data 1. Dotplots show individual data points and a line indicating their average value. Boxplot central line shows median value, box limits indicate the 25th and 75th percentile. Whiskers extend 1.5 times the interquartile range, or to the last datapoint. Individual datapoints are represented by dots

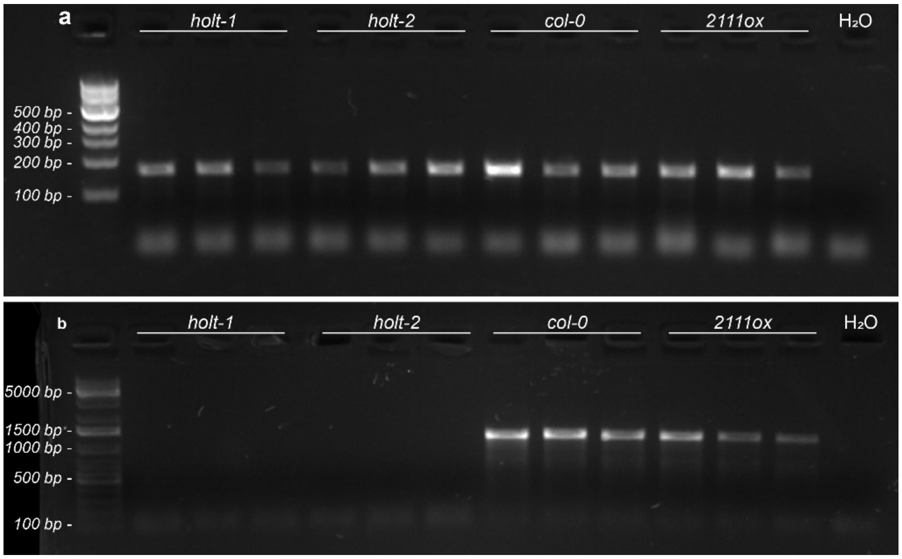

Supplementary Fig. 12. ***A. thaliana holt-1*** **and** ***holt-2*** **are putative knockout mutant lines showing no full-length** ***HOLT*** **transcript. a**,**b** Molecular characterization of the *holt-1* and *holt-2* mutants. **a** Amplicon of *HOLT* truncated transcript fragment 3' of the respective insertions, and 3' of F-Box as well as part of the Kelch-Repeat domain (Supplementary Fig. 8). **b** Fragments 5' of the insertion in *holt-1* cannot be generated from cDNA either of the *holt-1* or *holt-2* mutant lines, indicating absence of full-length *HOLT* transcripts in these mutants. **a-b**, Agarose gel electrophoresis of RT-PCR reactions. Col-0, wild type DNA positive control (ecotype Col-0); H_2O, negative control with water instead of DNA template added to reaction; 2111ox, *p35s::MIR2111b* plants. Two independent replicates showed the same results. All primers used for amplification are listed in Supplementary Table 5.

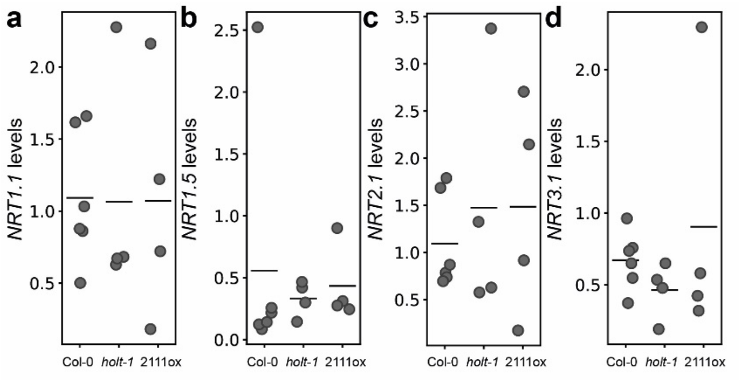

Supplementary Fig. 13. mRNA abundance of *A. thaliana NRT* genes is wild type-like in *holt-1* or *MIR2111b* overexpression plants. a-d Relative levels of *NRT1.1* (**a**), *NRT1.5* (**b**), *NRT2.1* (**c**) and *NRT3.1* (**d**) in *holt-1* and *p35s::MIR2111b* (2111ox) plants compared to ecotype col-0 wild type plants. Plants grown for ten days on 1 mM nitrate. **a-d** qRT-PCR analyses. RNA levels are relative to those of two reference genes. Comparisons using analysis of variance (ANOVA) identified no significant differences of mRNA abundances between lines. Sample size, replicates and exact p-values are listed in Supplementary Data 1. Dotplots show individual data points and a line indicating their average value.

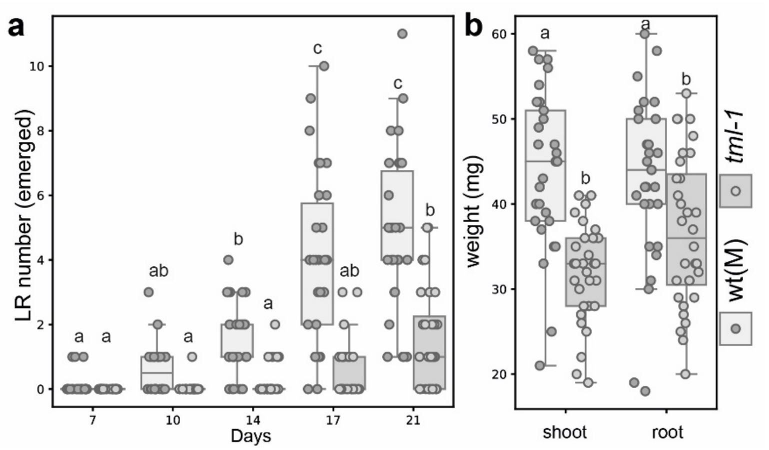

Supplementary Fig. 14. Root growth differences between *L. japonicus* (Lotus) *tml-1* and wild type increase over time. a Lateral root (LR) numbers of Lotus *tml-1* and wild type (ecotype MG20; wt(M)) plants at different timepoints between 7 and 21 days of cultivation. **b** Shoot and root weight of the same plants after four weeks of cultivation. Plants were grown at 1 mM nitrate. Comparisons used analysis of variance (ANOVA) and post-hoc Tukey testing ($p \leq 0.05$), with distinct letters indicating significant differences. Sample size, replicates and exact p-values are listed in Supplementary Data 1. Boxplot central line shows median value, box limits indicate the 25th and 75th percentile. Whiskers extend 1.5 times the interquartile range, or to the last datapoint. Individual datapoints are represented by dots.

Supplementary Information References

1 Klepikova, A. V., Kasianov, A. S., Gerasimov, E. S., Logacheva, M. D. & Penin, A. A. A high resolution map of the *Arabidopsis thaliana* developmental transcriptome based on RNA-seq profiling. *Plant J* **88**, 1058-1070, doi:10.1111/tpj.13312 (2016).

2 Devers, E. A. *et al.* Movement and differential consumption of short interfering RNA duplexes underlie mobile RNA interference. *Nat Plants* **6**, 789-799, doi:10.1038/s41477-020-0687-2 (2020).

3 Lu, Z. J., Gloor, J. W. & Mathews, D. H. Improved RNA secondary structure prediction by maximizing expected pair accuracy. *RNA* **15**, 1805-1813, doi:10.1261/rna.1643609 (2009).
