# Supplementary material for: A micro RNA mediates shoot control of root branching
Source: Nat Commun. 2023 Dec 6;14:8083. doi: 10.1038/s41467-023-43738-6 (PMC10700597; doi:10.1038/s41467-023-43738-6)
Supplement: Supplementary file 3 — Reporting Summary [file 41467_2023_43738_MOESM3_ESM.pdf]

nature portfolio

Corresponding author(s): Katharina Markmann

Last updated by author(s): Oct 9, 2023

Reporting Summary

Nature Portfolio wishes to improve the reproducibility of the work that we publish. This form provides structure for consistency and transparency in reporting. For further information on Nature Portfolio policies, see our Editorial Policies and the Editorial Policy Checklist.

Statistics

For all statistical analyses, confirm that the following items are present in the figure legend, table legend, main text, or Methods section.

n/a	Confirmed	
☐	☒	The exact sample size (*n*) for each experimental group/condition, given as a discrete number and unit of measurement
☐	☒	A statement on whether measurements were taken from distinct samples or whether the same sample was measured repeatedly
☐	☒	The statistical test(s) used AND whether they are one- or two-sided<br>*Only common tests should be described solely by name; describe more complex techniques in the Methods section.*
☐	☒	A description of all covariates tested
☒	☐	A description of any assumptions or corrections, such as tests of normality and adjustment for multiple comparisons
☒	☐	A full description of the statistical parameters including central tendency (e.g. means) or other basic estimates (e.g. regression coefficient) AND variation (e.g. standard deviation) or associated estimates of uncertainty (e.g. confidence intervals)
☒	☐	For null hypothesis testing, the test statistic (e.g. *F*, *t*, *r*) with confidence intervals, effect sizes, degrees of freedom and *P* value noted<br>*Give P values as exact values whenever suitable.*
☒	☐	For Bayesian analysis, information on the choice of priors and Markov chain Monte Carlo settings
☒	☐	For hierarchical and complex designs, identification of the appropriate level for tests and full reporting of outcomes
☒	☐	Estimates of effect sizes (e.g. Cohen's *d*, Pearson's *r*), indicating how they were calculated

Our web collection on statistics for biologists contains articles on many of the points above.

Software and code

Policy information about availability of computer code

Data collection	No software was used for data collection.
Data analysis	Secondary structure of Lotus japonicus MIR2111-3 wild type and ko precursor sequences was modelled using the MaxExpect algorithm with default settings. Reference: Lu, Z. J., Gloor, J. W. & Mathews, D. H. Improved RNA secondary structure prediction by maximizing expected pair accuracy. RNA 15, 1805-1813 (2009). https://doi.org:10.1261/rna.1643609.<br>Quantitative real-time PCR following reverse transcription (qRT-PCR) data analysis was supported by LinReg PCR using standard settings and the version published on 14.06.2021 (https://mybiosoftware.com/linregpcr-analysis-quantitative-pcr-data.html).

For manuscripts utilizing custom algorithms or software that are central to the research but not yet described in published literature, software must be made available to editors and reviewers. We strongly encourage code deposition in a community repository (e.g. GitHub). See the Nature Portfolio guidelines for submitting code & software for further information.

Data

Policy information about availability of data

All manuscripts must include a data availability statement. This statement should provide the following information, where applicable:

- Accession codes, unique identifiers, or web links for publicly available datasets
- A description of any restrictions on data availability
- For clinical datasets or third party data, please ensure that the statement adheres to our policy

All primary data and images analyzed in the context of this study are available from the corresponding author upon request.

Field-specific reporting

Please select the one below that is the best fit for your research. If you are not sure, read the appropriate sections before making your selection.

- [x] Life sciences
- [ ] Behavioural & social sciences
- [ ] Ecological, evolutionary & environmental sciences

For a reference copy of the document with all sections, see nature.com/documents/nr-reporting-summary-flat.pdf

Life sciences study design

All studies must disclose on these points even when the disclosure is negative.

Sample size	Following standard practices, experiments rely on a minimum of three biological replicates. Replicates and plant numbers (n) were increased until SD and SEM were sufficiently small to allow for meaningful comparative analyses using appropriate statistical tests.
Data exclusions	No data were excluded from the analyses.
Replication	Following standard practices, experiments rely on a minimum of three biological replicates. All presented data showed the same tendencies in all biological replicates performed. Data where results diverged between replicates are not shown, discussed or concluded from in the manuscript.
Randomization	Seedlings used for comparative analyses were pool-germinated and randomly allocated to respective treatments/conditions by technical personnel not aware of the experimental setup or working hypothesis.
Blinding	During phenotypic analyses, investigators were unaware of the respective genotype/treatment combinations they were investigating.

Reporting for specific materials, systems and methods

We require information from authors about some types of materials, experimental systems and methods used in many studies. Here, indicate whether each material, system or method listed is relevant to your study. If you are not sure if a list item applies to your research, read the appropriate section before selecting a response.

Materials & experimental systems

n/a	Involved in the study	
x		Antibodies
x		Eukaryotic cell lines
x		Palaeontology and archaeology
	x	Animals and other organisms
x		Human research participants
x		Clinical data
x		Dual use research of concern

Methods

n/a	Involved in the study	
x		ChIP-seq
x		Flow cytometry
x		MRI-based neuroimaging

Animals and other organisms

Policy information about studies involving animals; ARRIVE guidelines recommended for reporting animal research

Laboratory animals	The study did not involve laboratory animals.
Wild animals	The study did not involve wild animals.
Field-collected samples	The study did not involve samples collected from the field.
Ethics oversight	No ethical approval or guidance was required, as our work exclusively relied on plant and bacterial organisms.

Note that full information on the approval of the study protocol must also be provided in the manuscript.
