# Supplementary figures and images for: A micro RNA mediates shoot control of root branching
Source: Nat Commun. 2023 Dec 6;14:8083. doi: 10.1038/s41467-023-43738-6 (PMC10700597; doi:10.1038/s41467-023-43738-6)

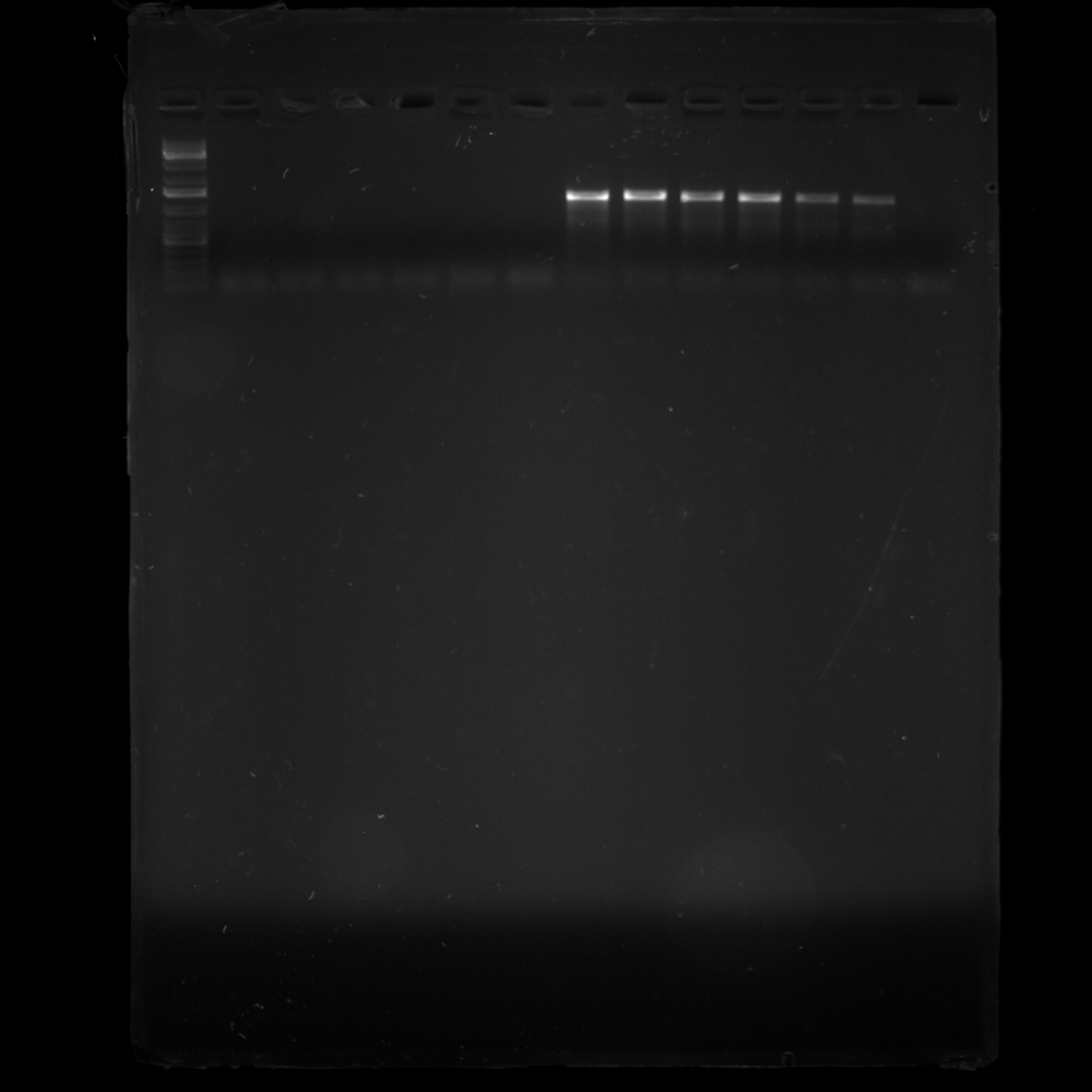

Supplement: Supplementary file 4 — Source Data [file 41467_2023_43738_MOESM4_ESM.zip › Source data Supplementary Figure 12a.Tif]

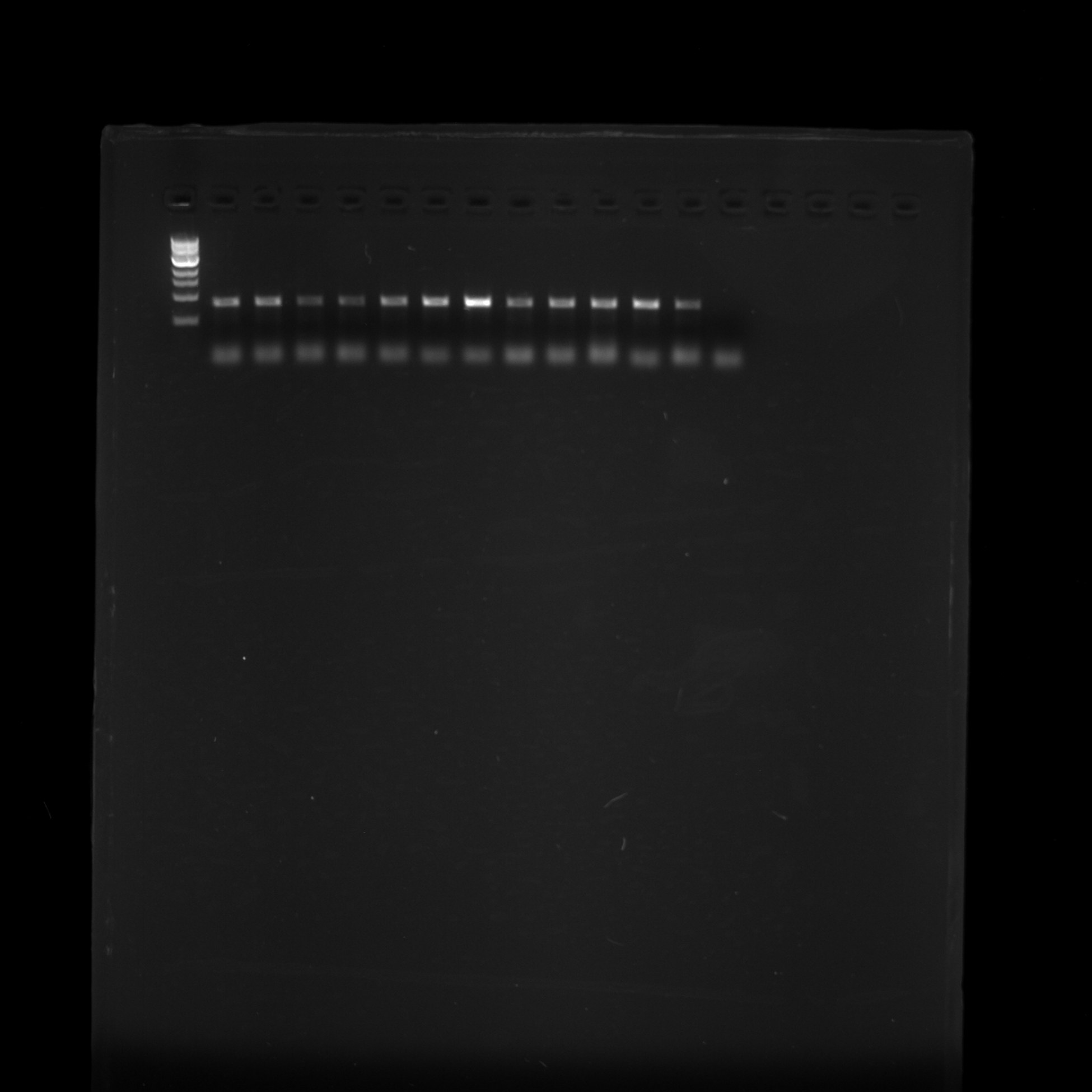

Supplement: Supplementary file 4 — Source Data [file 41467_2023_43738_MOESM4_ESM.zip › Source data Supplementary Figure 12b.Tif]
